# Prediction of new Hsp90 inhibitors based on 3,4-isoxazolediamide scaffold using QSAR study, molecular docking and molecular dynamic simulation

**DOI:** 10.1186/s40199-017-0182-0

**Published:** 2017-06-30

**Authors:** Maryam Abbasi, Hojjat Sadeghi-Aliabadi, Massoud Amanlou

**Affiliations:** 1 Department of Medicinal Chemistry, Faculty of Pharmacy, Isfahan University of Medical Sciences, Isfahan, 81746-73461 Iran; 2 Computational Chemistry Group, Pharmaceutical Sciences Research Center and Department of Medicinal Chemistry, Faculty of Pharmacy, Tehran University of Medical Sciences, Tehran, Iran

**Keywords:** Hsp90, Inhibitor, 3,4-Isoxazolediamide, QSAR, Molecular docking, Molecular dynamic simulation

## Abstract

**Background:**

Heat shock protein90 (Hsp90) are overexpressed in tumor cells, so the inhibition of the Hsp90 ATPase activity would be a significantly effective strategy in cancer therapy.

**Methods:**

In the current study, 3,4-isoxazolediamide derivatives were suggested as an Hsp90 inhibitor for anti-cancer therapy. Multiple linear regression (MLR) and genetic algorithm of partial least square (GA-PLS) methods were performed to build models to predict the inhibitory activity of Hsp90. The leave-one out (LOO) cross-validation and Y-randomization tests were performed to models’ validation. The new ligands were monitored by applicability domain. Molecular docking studies were also conducted to evaluate the mode of interaction of these compounds with Hsp90. Identification of the likely pathways into the active site pocket and the involved residues were performed by CAVAER 3.0.1 software. According to QSAR models and docking analysis, three new compounds were predicted. 50 ns molecular dynamic simulation was performed for the strongest synthesized compound and the best predicted compound in terms of binding energy and interactions between ligand and protein.

**Results:**

The made models showed the significance of size, shape, symmetry, and branching of molecules in inhibitory activities of Hsp90. Docking studies indicated that two hydroxyl groups in the resorcinol ring were important in interacting with Asp93 and the orientation of these groups was related to substitution of different R_1_ groups. Comparing of molecular dynamic simulation (MDs) results shows that new compound perched in active site with lower binding energy than the best synthesized compound.

**Conclusion:**

The QSAR and docking analyses shown to be beneficial tools in the proposal of anti-cancer activities and a leader to the synthesis of new Hsp90 inhibitors based 3,4-isoxazolediamide. The MDs confirmed that predicted ligand is steady in the Hsp90 active sites.

**Graphical Abstract:**

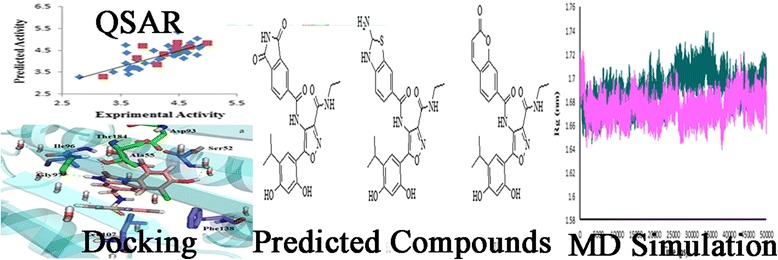

## Background

**Fig. 1:**
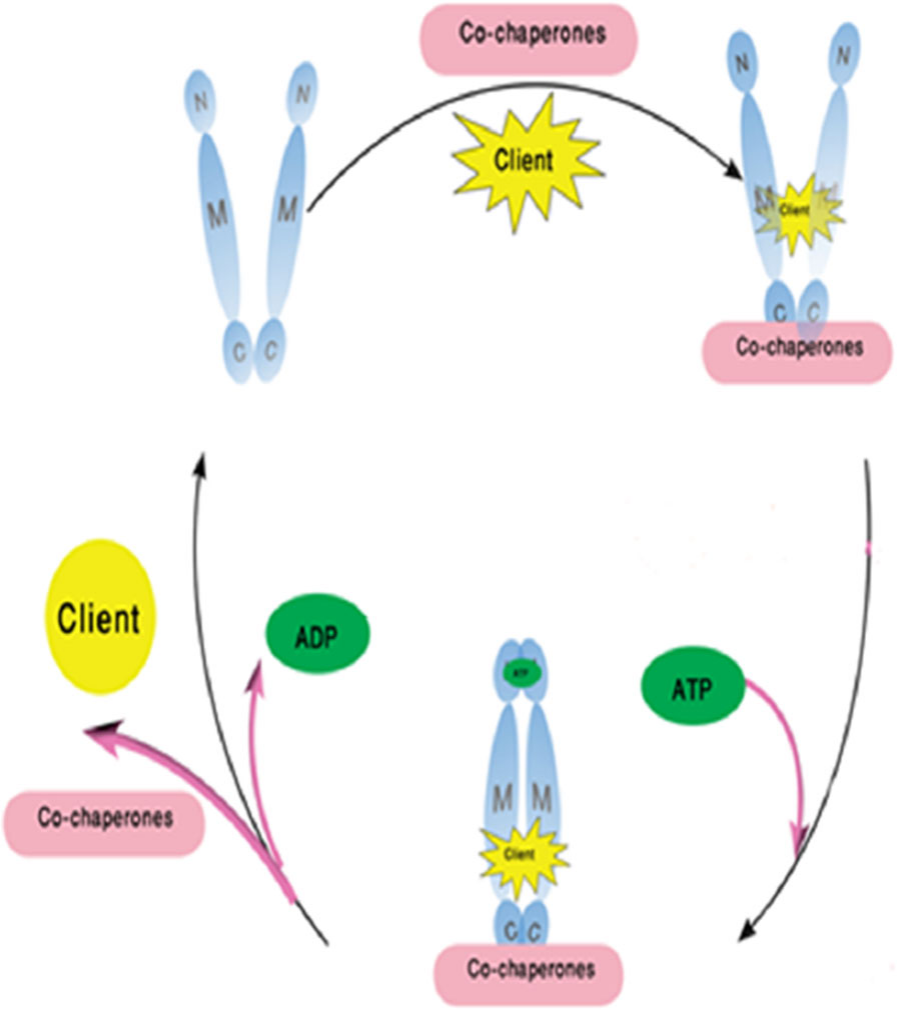
ATPase cycle in Hsp90. The first, in open state of protein, co-chaperones and client protein bind to C-terminal and middle domain, respectively. ATP bind to N-terminal and Hsp90 is closed. Then ATP is hydrolyzed and the complex changed. Finally, client protein is folded

Heat shock proteins (Hsps) play a critical role in maturation and stabilization of proteins in the cell [1]. One of the important Heat shock proteins in cell is Hsp90. The Hsp90 molecular chaperone contributes to folding of more than 200 proteins (client proteins) and it is necessary for adjusting the balance between the synthesis and degradation of many proteins in the cell [2, 3]. The homo dimer of Hsp90 possess three main domains: the N-terminal domain that contains the nucleotide-binding pocket, the middle region that is involved in binding of client proteins and the C-terminal domain that is the dimerization site [4]. The Hsp90 function is reliant on its ability to bind and hydrolyze ATP at the N-terminal domain. First, client protein and co-chaperones bind to Hsp90 in the open condition of protein and then ATP binds to N-terminal and Hsp90 will be closed. Finally, ATP is hydrolyzed, the complex is changed and client protein is folded (Fig. 1) [5].

**Fig. 2:**
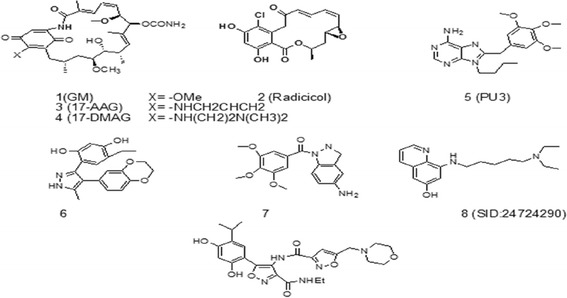
Several categories of Hsp90 inhibitors. Natural inhibitors (geldanamycin, GM, (**1**), radicicol (**2**)), reclaimed analogues of GM (17-AAG (**3**) and 17-DMAG (**4**)), synthetic inhibitors (purine (**5,** PU3), pyrazole (**6**), indazole (**7**), aminoquinolines (**8,** SID: 24724290) and isoxazole (**9**))

**Fig. 3:**
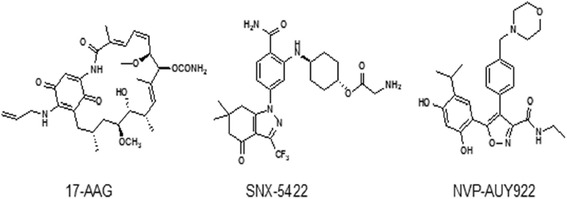
The structure of three clinically Hsp90 inhibitors evaluated in human. Derivative of geldanamycin (17-AAG), carbazol-4-one benzamide (SNX-5422) and isoxazole (NVP-AUY922)

In tumor cells, Hsp90 is overexpressed and causes the uncontrolled proliferation of transformed cells [6], so inhibition of the Hsp90 ATPase activity can be a significantly effective strategy in cancer therapy [7]. Hsp90 inhibitors are classified into several categories containing natural inhibitors (geldanamycin, GM (1), and radicicol (2)), reclaimed analogues of GM (17-AAG (3) and 17-DMAG (4), synthetic inhibitors (purine (PU3 (5)), pyrazole (6), indazole (7), aminoquinolines (SID: 24724290 (8)) and isoxazole (9) that are shown in Fig. 2 [8–11]. Some of these inhibitors, such as the reclaimed analogue of geldanamycin (17-AAG), carbazol-4-one benzamide derivative (SNX-5422) and isoxazole derivative (NVP-AUY922, currently known as Luminespib), have been assessed in humans (Fig. 3) [1]. Among the different azaheterocyclic ring systems, the isoxazole scaffold is one of the most promising heterocyclic systems [12]. Baruchello and coworkers in 2011 synthesized novel 3, 4-isoxazolediamides as potent inhibitors of Hsp90 [8].

Forasmuch as obtaining a new inhibitor requires much time and capital, any tool that can help to precipitate the drug development processes would be very noteworthy [13]. The advanced computational techniques are highly useful strategies to conduct rapid and inexpensive investigations on large databases and obtaining new inhibitors [14].

Molecular docking, molecular dynamic simulation and quantitative structure activity relationships (QSARs) are helpful computational methods for drug design and activity prediction [15, 16]. In molecular docking and molecular dynamic simulation, the 3D structure of the receptor will be available and receptor-ligand interactions play an important role, so this drug design is called structure-based drug design [17]. Docking is a method which proposes the favored orientation and energy of one ligand when bound in the active site to build a stable complex. To investigate interactions between ligand and protein, molecular dynamic simulation will be performed.

QSAR is referred to as ligand-based drug design because it is performed based on the knowledge about ligands [18]. QSAR models are mathematical equations that create relationships between chemical structures and their biological activities without considering a receptor.

In the present study, we performed a QSAR study for modeling the inhibitory effects of 50 synthesized 3,4-isoxazolediamides [8]. Multiple linear regression (MLR) and genetic algorithm of partial least square (GA-PLS) methods were used for modeling the relationship between pIC_50_ and their structural descriptors. Molecular docking was performed for 25 compounds and the results were compared with experimental data. According to QSAR models and molecular docking analysis three compounds were predicted. Molecular dynamic simulation method was chosen to compare the best synthesized and the best predicted compound in terms of binding energy and interactions between protein and ligand.

## Methods

### Activity data and generation of descriptors

In the current study, the Hsp90 inhibitory activity of 50 compounds that were synthesized and evaluated by Baruchello and coworkers, were used as the biological data [8]. To make the mistake less, the compounds were chosen from an article with the same assay. Reported compounds were chosen from isoxazole derivatives. The lead of these compounds was NVP-AUY922 (currently known as Luminespib) that was entered into clinical trials and many of its derivatives were synthesized up to now. These compounds are shown in Table 1.

**Table 1:**
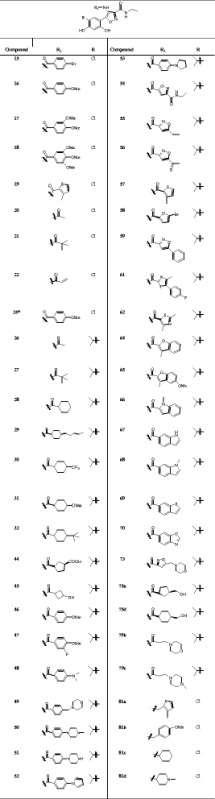
Chemical structures of 3, 4-isoxazolediamides derivatives as Hsp90 inhibitors

*NHCH_2_CH_2_ converted to NHCH_2_CF_3_

Geometry optimization was performed by two methods: MM^+^ molecular mechanic force field and the semi-empirical PM3 by HyperChem 8.0 software. Both optimizations were carried out by the Polak–Ribiere algorithm until the root mean square gradient reached 0.01 kcal/Å mol. Chemical descriptors were also generated by HyperChem software.

HyperChem outputs were conveyed to DRAGON package to compute four classes of descriptors: 0D, 1D, 2D and 3D. Different quantum chemical descriptors were obtained by Gaussian98 program [19]. All of the calculated descriptors are shown in Table 2.

**Table 2:** Brief of used descriptors in this study

Descriptor type	Molecular description
Chemical	Surface area, molecular volume, hydration energy, octanol/water partition coefficient (logP), molar refractivity, molar polarisability and molar mass.
0D,1D,2D and 3D	28 constitutional descriptors, 10 functional groups, 18 atom-centered fragments, 216 topological descriptors, 15 molecular walk counts, 64 BCUT descriptors, 24 Galvestopol. charge indices, 96 2D autocorrelations, 14 charge descriptors, 41 Randic molecular profiles, 27 geometrical descriptors, 150 radial distribution function descriptors (RDF), 160 3D–MoRSE descriptors, 99 WHIM descriptors and 196 GETAWAY descriptors
Quantum chemical	Highest occupied molecular orbital energy (EHOMO), lowest unoccupied molecular orbital energy (ELUMO), molecular dipole moment and local charge were obtained with PM3 method in Gaussian 98. Hardness (η = 0.5 (EHOMO + ELUMO)), softness (S = 1/η), electronegativity (χ = 0.5 (EHOMO - ELUMO)) and electrophilicity (ω = χ2/2η)

### Data processing and model building

All of the calculated descriptors were used to generate a 50 × 1126 data matrix; the number on its rows is illustrative of molecule numbers and the numbers on columns accounts for descriptors. The columns which had constant and near constant amounts were deleted from this data matrix. Since as the models were disrupted by collinear variables, collinear descriptors had to be found and eliminated and then descriptors’ correlations with one another and with activity data were checked. In couples with collinearities higher than 0.9, the highest correlation with the activity was kept and the rest were eliminated. The number of total descriptors for each molecule attained 352.

The data set (50 compounds) was divaricated into the calibration set and validation set. Validation subset was prepared from 20% of the total data (here, 9 biological activity data). The model was made by MLR analysis, with the stepwise regression SPSS (Version 12.0) software.

The combined data splitting feature selection (CDFS) approach was utilized because the data splitting has a significant effect on the terminal selected model [15]. In the CDFS approach, numerous subdivisions of the calibration and validation set were created (5 times). In each case, the best model was elected. The chosen models were validated by leave-one out (LOO) cross-validation method. According to 5 models descriptors, general model was created and Y-randomization test was performed to check their predictability.

### Molecular docking

Molecular docking of 3,4-isoxazolediamide derivatives (25 compounds) as Hsp90 inhibitors was perused by AutoDock 4.2 program to detect their binding site, the best direction and the binding energy [20]. Between the experimental X-ray structures of Hsp90, the crystallography structure with a PDB entry code of 3OWD was chosen [21].

For protein preparation, the co-crystallized ligand and water molecules, except the water molecules that were significant in interaction between the ligand and protein, were deleted. By AutoDockTools, all missing hydrogens were added. The Kollman atom charges were calculated, non-polar hydrogens merged and the file saved as pdbqt. A grid box was created with a grid point spacing of 0.375 Å and 90 × 90 × 90 points, which included not only the active site of the protein but also significant regions of the surrounding surface. Before calculating the grid maps by AutoGrid 4.2 [22], the parameters of the water molecules were added to AD4-bound and AD4-parameter files.

After the ligands were prepared, the 3D structures of all compounds were dragged in Marvin Sketch Ver. 5.7, ChemAxon [23]. The partial charges of atoms were computed by the Gasteiger–Marsili procedure and non-polar hydrogens of the compounds were merged [24].

The Lamarckian genetic algorithm approach was elected for the global optimum binding position. Docking computing parameters were determined as the following: number of Lamarckian job = 50, initial population = 150, maximum number of energy evaluation = 25 × 10^5^, and the default values of other parameters were kept unchanged. The docking parameter file (.dpf) was created. The docking procedure was done by AutoDock 4.2 and the .dlg file was generated. All of the runs were ranked by the maximum number of clusters and the lowest binding energy and were analyzed to find the best conformation of the ligand with key residues in the active site of the protein by Accelrys Discovery Studio 2.5 [25] and PyMOL software [26].

### Caving active site tunnels

To monitor likely pathways into the active site pocket of the protein and also recognize the involved residues, CAVAER 3.0.1 software was employed [27]. Likely entrances were searched by assigning maximum probe radius to 0.9Ǻ, shell depth to 4, and clustering threshold to 3.5Ǻ.

### Molecular dynamic simulation

Molecular dynamic simulation was carried out with the GROMACS 5.0.5 package [28]. The topology parameters of the best predicted ligand in terms of the energy and interaction between ligand and protein were created by the PRODRG web server [29]. The generated charges by this server were corrected by Gaussian98 program. The pKa for residues of protein were obtained by the PROPKA 3.1 web server to determined which residue was more possible to embrace nonstandard ionization states [28]. The key crystallographic water molecules in the active site were kept [25]. The GROMOS96 54a7 force field and the simple point charges (SPC) water model were used to create protein topology parameters. The complex of ligand and protein was dunked in dodecahedron box with a minimum distance of 1 nm between the protein surface and box border, containing of about 8250 solvent molecules. By displacing solvent water molecules with 4 Na + was neutralized the system net charge. The energy minimization was done to release spatial clashes of the complex in two steps. First, only water molecules were minimized by using 10,000 steepest descents steps while the other atoms were hold fixed at their initial configuration. After that, the entire system was minimized. To equilibrate the system at a constant temperature of 300 K was performed NVT step by a 500 ps MD run. After the stabilization of temperature by the V-Rescale algorithm, an NPT ensemble was done with time duration of 1 ns. This was followed by MD production run at 1 bar pressure and 100, 200 and 300 K temperatures for 1, 2 and 50 ns, respectively. Long-range electrostatic interactions were computed with the Particle Mesh Ewald (PME) method. The linear constraint (LINCS) algorithm was used for covalent bond constraints. Structure visualization was carried out by VMD 1.8.6 and PyMOL.

## Result and discussion

### Multiple linear regression analysis (MLR)

Stepwise regression method was used to select the most appropriate set of descriptors for each type of the split data. Models were selected; of course, some of them could be over-fitted. Another method which could be utilized to select the model or the most suitable correlation equation was the cross-validation method. In this case, the obtained models were evaluated for over-fitting by leave-one-out cross-validation (LOO) method and then were consistently graded for cross-validation (Q^2^_LOO_) by the square correlation coefficient. Eventually, one model with equilibrium between the highest R^2^_c_ and Q^2^_LOO_ was chosen for future analysis.

MLR method was performed five times by distinct split data. In each case, one model was offered. The best five models are reported in Table 3. The models demonstrate high statistical qualities. All of the models have Q^2^_LOO_ greater than 0.5; hence, the expected models indicated suitable results for the prediction set. The values of prediction correlation coefficient (R^2^_p_) for the five final models are documented in Table 3.

**Table 3:** The best five models were selected for future analysis

NO.	Descriptors used	R^2^_c_^a^	S.E^b^	R^2^_p_^c^	Q^2 d^	RMS_CV_^e^
1	X5A, HATS4u, Mor10m, Mor26p, Mor09u, Du	0.806	0.249	0.958	0.723	0.276
2	BELe1, MATS6e, Gu, Mor27p, Mor12m, RDF140m	0.760	0.266	0.817	0.586	0.329
3	X5A, T (N..O), dipole y, R3e+, R1e, Mor09m	0.768	0.214	0.911	0.683	0.254
4	X5A, HATS4u, Mor26p, TIE, dipole z, Mor26e, ISH	0.814	0.245	0.883	0.723	0.274
5	X5A, T (N..O), MATS1p, dipole z, MATS8e, Ku, P2m	0.790	0.252	0.944	0.696	0.278

^a^R^2^_c_ = Correlation Coefficient of calibration set. ^b^S.E = Standard error of regression. ^c^R^2^_p_ = Correlation Coefficient of prediction set. ^d^Q^2^ = Leave-one-out cross-validation correlation coefficient. ^e^RMSE_CV_ = Root mean square error of cross validation

The total number of descriptors that existed in all five models was 25. These descriptors are briefly defined in Table 4. Among these, X5A and 3D–MoRSE descriptors have been repeated in four models. This means that connectivity indices (X5A) and 3D–MoRSE descriptors have main effect on Hsp90 inhibitors which are based on 3, 4-isoxazolediamide scaffold. Some of the descriptors such as Du, BELe1, RDF140m, Gu, ISH, Ku, P2m, and TIE were observed only in one model, and have a lower effect on Hsp90 inhibitors.

**Table 4:** Brief description of the descriptors in five models

NO.	Name	Description
1	X5A	Connectivity indices-average connectivity index of order 5.
2	HATS4u	GETAWAY descriptors-leverage-weighted autocorrelation of lag 4 / unweighted.
3	Mor10m	3D–MoRSE descriptors-signal 10 / weighted by mass.
4	Mor26p	3D–MoRSE descriptors-signal 26 / weighted by polarizability.
5	Mor09u	3D–MoRSE descriptors-signal 09 / unweighted.
6	Du	WHIM descriptors-D total accessibility index / unweighted.
7	BELe1	Lowest eigenvalue n. 1 of Burden matrix / weighted by atomic Sanderson electronegativities.
8	MATS6e	Moran Autocorrelation-lag 6/weighted by atomic Sanderson electronegativities.
9	Gu	WHIM descriptors-total symmetry index / unweighted.
10	Mor27p	3D–MoRSE descriptors-signal 27 / weighted by polarizability.
11	Mor12m	3D–MoRSE descriptors-signal 12 / weighted by mass.
12	RDF140m	Radial Distribution Function-140 / weighted by mass.
13	T (N..O)	2D Atom Pairs-sum of topological distances between N..O.
14	Dipole y	An electric dipole is located along the y axis.
15	R3e+	GETAWAY descriptors-R maximal autocorrelation of lag 3 / weighted by Sanderson electronegativity.
16	R1e	GETAWAY descriptors-R autocorrelation of lag 1 / weighted by atomic Sanderson electronegativities
17	Mor09m	3D–MoRSE descriptors-signal 09 / weighted by mass.
18	TIE	Topological indices-E-state topological parameter.
19	Dipole z	An electric dipole is located along the z axis.
20	Mor26e	3D–MoRSE descriptors-signal 26 / weighted by Sanderson electronegativity.
21	ISH	GETAWAY descriptors-standardized information content on the leverage equality.
22	MATS1p	Moran autocorrelation - lag 1 / weighted by atomic polarizabilities
23	MATS8e	Moran Autocorrelation-lag 8/weighted by atomic Sanderson electronegativities.
24	Ku	K global shape index / unweighted
25	P2m	2nd component shape directional WHIM index / weighted by atomic masses

To generate a general model for split data, all of the 25 descriptors were applied and MLR analysis was conducted with the stepwise variable selections. The resulted model is reported in MLR equation:

$$ {\mathrm{pIC}}_{50}=16.977\left(\pm 1.966\right)-176.806\left(\pm 28.236\right)\ \mathrm{X}5\mathrm{A}-4.366\left(\pm 1.046\right)\ \mathrm{HATS}4\mathrm{u}-1.951\left(\pm 0.549\right)\ \mathrm{Mor}26\mathrm{p}+0.001\left(\pm 0.00\right)\ \mathrm{TIE}+0.227\left(\pm 0.069\right)\ \mathrm{dipole}\ \mathrm{z}+0.863\left(\pm 0.368\right)\ \mathrm{Mor}26\mathrm{e} $$

$$ \left(\mathrm{N}=41,{{\mathrm{R}}^2}_{\mathrm{c}}=0.771,\mathrm{S}.\mathrm{E}=0.267,{{\mathrm{Q}}^2}_{\mathrm{LOO}}=0.637,{\mathrm{R}\mathrm{MS}}_{\mathrm{CV}}=0.297\right) $$

Where N demonstrates the number of molecules used in the calibration set. R^2^_c_ and Q^2^_LOO_ are respectively the squared correlation coefficient for calibration and cross-validation. In addition, S.E is standard error of calibration and RMS_CV_ is root mean square error of cross-validation.

In this equation, X5A, HATS4u and Mor26p have a minus, which indicates that the pIC_50_ is inversely related to these descriptors. Among these, X5A has a main effect on 3, 4-isoxazolediamide Hsp90 inhibitors. Connectivity indices, such as X5A, are among topological indices that are numerical quantifiers of molecular topology and an H-depleted molecular graph. They involve one or more structural features of the molecule such as the size, shape, symmetry, and branching and can also codify chemical information about atom type and bond multiplicity. 3D–MoRSE descriptors, such as Mor26p, illustrate three-dimensional coordination of the different atoms in the molecule. Geometry, topology, and atom-weights assembly (GETAWAY) descriptors, such as HATS4u, have been proposed as chemical structure descriptors.

TIE and dipole z display positive signs, which indicates the pIC_50_ is straight related to these descriptors. Topographic indices such as TIE establish a special subset of geometrical descriptors, being computed on the graph illustration of molecules but using the geometric distances between atoms instead of the topological distances [30].

**Fig. 4:**
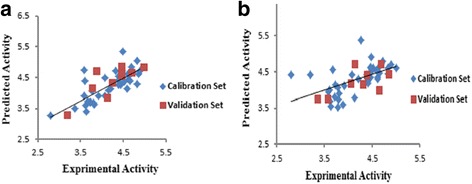
**a** Plot of predicted pIC_50_ versus the experimental values for MLR model, **b** GA-PLS model

The general model has a Q^2^_LOO_ equal to 0.710; hence, the predicted model can construct over 71% of variances in the inhibitory activity. The proposed values of pIC_50_, which were calculated for all the molecules by the MLR equation, along with the experimental pIC_50_ are listed in Table 5 and the predicted values of pIC_50_ are plotted against the experimental values in Fig. 4a.

**Table 5:** Experimental pIC_50_ and MLR and GA-PLS predicted pIC_50_

NO.	Experimental pIC_50_	MLR pIC_50_	GA-PLS pIC_50_	NO.	Experimental pIC_50_	MLR pIC_50_	GA-PLS pIC_50_
15	3.82	3.64	3.89	53	4.66	4.59	3.96
16	3.74	3.71	4.13	54	4.62	4.65	4.57
17	3.79	4.13	3.52	55	4.70	4.84	4.70
18	3.89	4.69	3.57	56	4.72	4.68	4.79
19	4.13	3.84	3.78	57	4.08	4.48	4.41
20	4.43	4.25	4.29	58	4.66	4.39	4.47
21	4.07	3.91	4.15	59	3.60	4.38	3.99
22	3.78	4.06	4.06	61	4.40	4.41	4.30
24	3.80	3.67	3.74	62	5.00	4.82	4.61
26	4.26	4.32	5.39	64	3.59	4.75	3.95
27	4.39	4.55	4.41	65	4.32	4.72	4.15
28	3.72	3.70	3.81	66	4.21	4.14	4.30
29	3.58	3.90	3.73	67	4.48	4.84	4.90
30	3.62	3.60	3.68	68	4.47	4.62	4.61
31	4.62	4.44	4.33	69	4.70	4.64	4.73
32	4.52	4.37	4.61	70	4.82	5.04	4.44
44	4.41	4.54	4.18	73	4.40	4.26	4.39
45	4.85	4.60	4.42	75a	4.90	4.72	4.69
46	4.58	4.14	4.52	75d	4.82	4.29	4.61
47	4.47	4.54	4.62	79b	3.67	3.72	4.55
48	4.47	4.72	4.34	79c	3.92	3.92	4.11
49	4.15	4.08	4.68	81a	3.64	3.41	3.54
50	4.62	4.60	4.30	81b	3.37	3.49	3.74
51	4.49	4.52	4.53	81c	3.21	3.28	4.42
52	4.49	5.36	4.56	81d	2.80	3.26	4.42

The robustness of the general model was evaluated by the Y-randomization test and 10 models were created. In a suitable MLR model, the values of R^2^ and Q^2^_LOO_ are lower than the original MLR model. The lower R^2^ and Q^2^_LOO_ values are shown in Table 6.

**Table 6:** R^2^ and Q^2^_LOO_ values after ten Y-randomization tests in MLR and GA-PLS

Iteration	MLR		GA-PLS	
	R^2^	Q^2^_LOO_	R^2^	Q^2^_LOO_
1	0.158	0.021	0.069	0.081
2	0.034	0.162	0.096	0.022
3	0.186	0.025	0.140	0.004
4	0.134	0.013	0.080	0.082
5	0.085	0.006	0.115	0.001
6	0.102	0.001	0105	0.016
7	0.119	0.001	0.182	0.018
8	0.082	0.032	0.072	0.054
9	0.075	0.047	0.185	0.004
10	0.091	0.004	0.082	0.024

### Genetic algorithm partial least squares (GA-PLS)

In addition to MLR method, the stepwise regression, a variable selection method, such as genetic algorithm, was performed. In genetic algorithm, if its related descriptor is comprised, a gene takes a value of 1 in the subset; otherwise, it receipts a value of zero. The number of genes is corresponding to the number of descriptors.

In GA-PLS study, the total number of descriptors for each molecule attained 117 and the highest fitness was selected as the proposed model. The intended model was tested by leave-n-out cross-validation [31]. A leave-3-out cross-validation was generated and Q^2^_LTO_ value of 0.834 could be established.

The eventuated model with higher cross-validation statistics is shown in the following equation and the predicted values of pIC_50_ are reported in Table 5 and plotted against the experimental values in Fig. 4b.

$$ \mathrm{pIC}50=9.018\left(\pm 2.799\right)-114.521\left(\pm 30.435\right)\ \mathrm{X}5\mathrm{A}-1.406\left(\pm 0.266\right)\ \mathrm{G}\mathrm{ATS}4\mathrm{e}-1.586\left(\pm 0.991\right)\ \mathrm{E}3\mathrm{u}-2.615\left(\pm 0.649\right)\ \mathrm{MATS}7\mathrm{e}+38.705\left(\pm 8.531\right)\ \mathrm{G}1\mathrm{u}+0.048\left(\pm 0.014\right)\ \mathrm{RDF}075\mathrm{m} $$

$$ \left(\mathrm{N}=41,{\mathrm{R}}^2\mathrm{c}=0.755,\mathrm{S}.\mathrm{E}=0.249,{{\mathrm{Q}}^2}_{\mathrm{LOO}}=0.653,\mathrm{RMSCV}=0.266\right) $$

In this equation, X5A descriptor, like MLR equation, has a minus and also has a main effect on 3, 4-isoxazolediamide Hsp90 inhibitors. GATS4e, E3u, and MATS7e also have minuses. Unlike these, G1u and RDF075m (WHIM and the radial distribution function (RDF) descriptors) have a positive efficacy on GA-PLS.

The created model in GA-PLS was evaluated by Y-randomization test. The R^2^ and Q^2^_LOO_ values of the ten created models were lower than the original GA-PLS model, as shown in Table 6.

### Applicability domain of the model

**Fig. 5:**
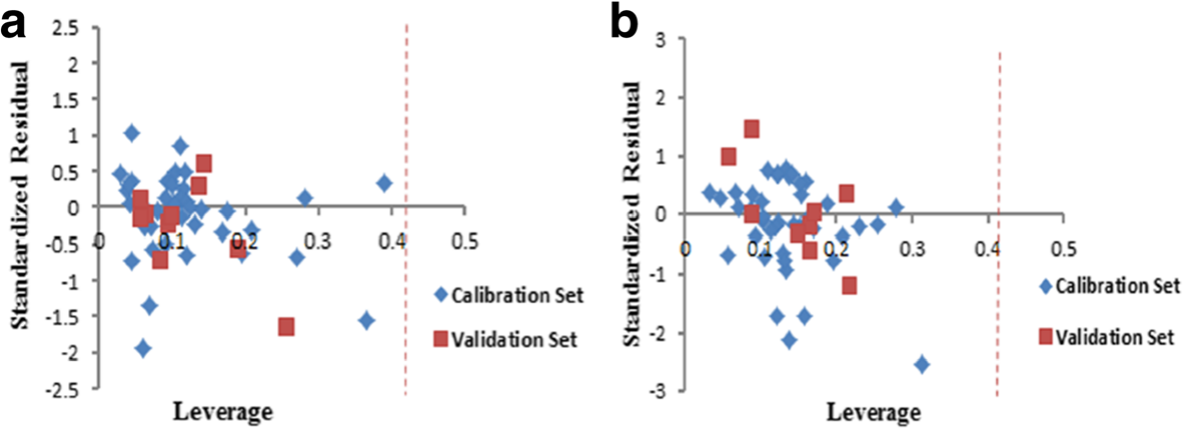
**a** William’s plot of generated MLR model, **b** GA-PLS model

A QSAR model is applied to monitor new compounds when its domain of application has been determined [29]. The divination may be supposed valid for only those compounds which fall into this domain [15]. Therefore standardized residuals of the activity were calculated and plotted against leverage values (h). The value of leverage was computed for every compound. Values are evermore between 0 and 1. A value of 1 indicates very poor prediction, and a value of 0 is indicative of perfect prediction and is not usually available. The lower the value is, the higher the assurance in the prediction. Warning leverage (h*) is another standard for description of the results and is commonly stabilized at 3 (k + 1)/n, where k is the number of model parameters and n is the number of calibration set [15]. Obtained leverage for calibration set is used to define the compounds which affect the model and, in terms of validation set, used to determine the applicability domain of the model. William’s plot for the advanced models in MLR and GA-PLS is revealed in Fig. 5.

Rejoinder outliers are compounds that have standard residual points higher than ±2.5 and ±3.0 standard deviation units for MLR and GA-PLS respectively and also a leverage value higher than the warning leverage, which is 0.42. Figure 5 shows that all the considered molecules in calibration and validation set lie with a high degree of assurance in the application domain of the developed models in both methods.

### Molecular docking

ATP is Hsp90 substrate and its active site includes mixed hydrophobic, polar and charged amino acids. These residues include Leu48, Asn51, Asp54, Ala55, Lys58, Ile91, Asp93, Ile96, Gly97, Met98, Asn106, Leu107, Lys112, Gly135, Phe138, Val150, Thr184, and Val186. While the pocket becomes increasingly hydrophobic toward the bottom, one charged residue and one polar residue are retained as Asp93 and Thr184 respectively [32]. The adenine ring in ATP sits at the bottom of the pocket and its N6 group form direct hydrogen bonds with Asp93 (Asp79 in yeast Hsp83), and water-mediated hydrogen bonds with the Ser52 and Leu48. Also, Glu47 (Glu33 in yeast Hsp83) gets involved in Hsp90 ATPase activity [33, 34].

**Fig. 6:**
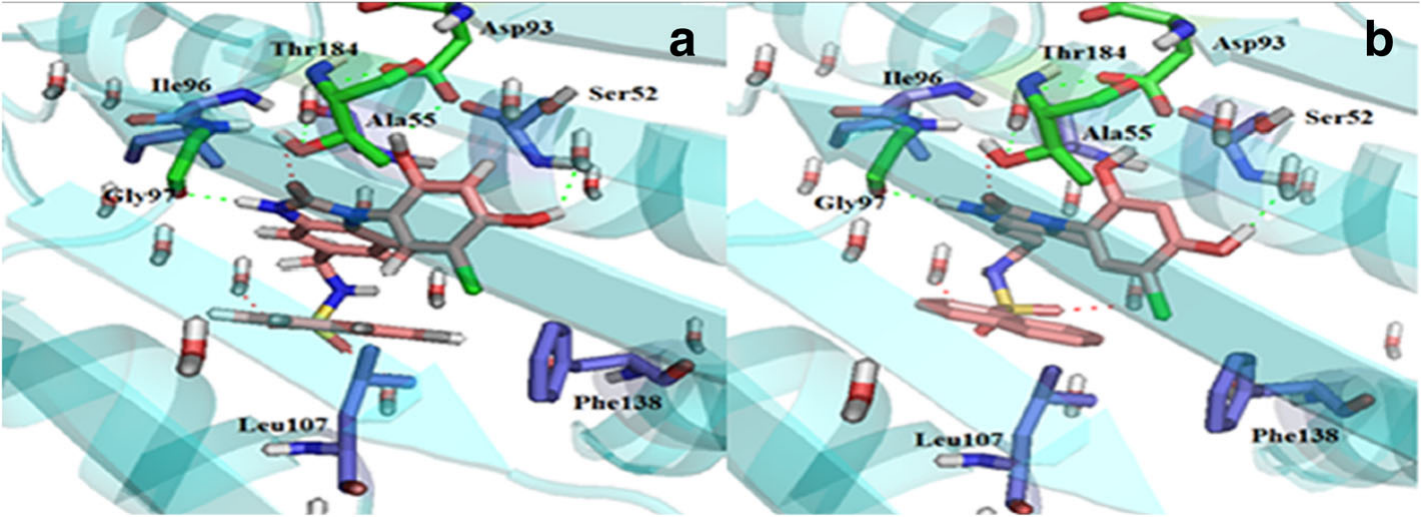
**a** The position of ligand in X-ray crystallography, **b** The position of ligand after redocking

A crystallography structure of 3OWD was chosen for the structure-based drug design. A validation of docking procedure was done by the extraction of the ligand from X-ray complex and redocking it. A .dlg file was created. A cluster root-mean-square deviation (RMSD) at less than 2 Å whose initial coordinates of the ligand were used as the reference structure was observed. The obtained results show that the docked ligand was located in the active site of Hsp90 (Fig. 6).

**Fig. 7:**
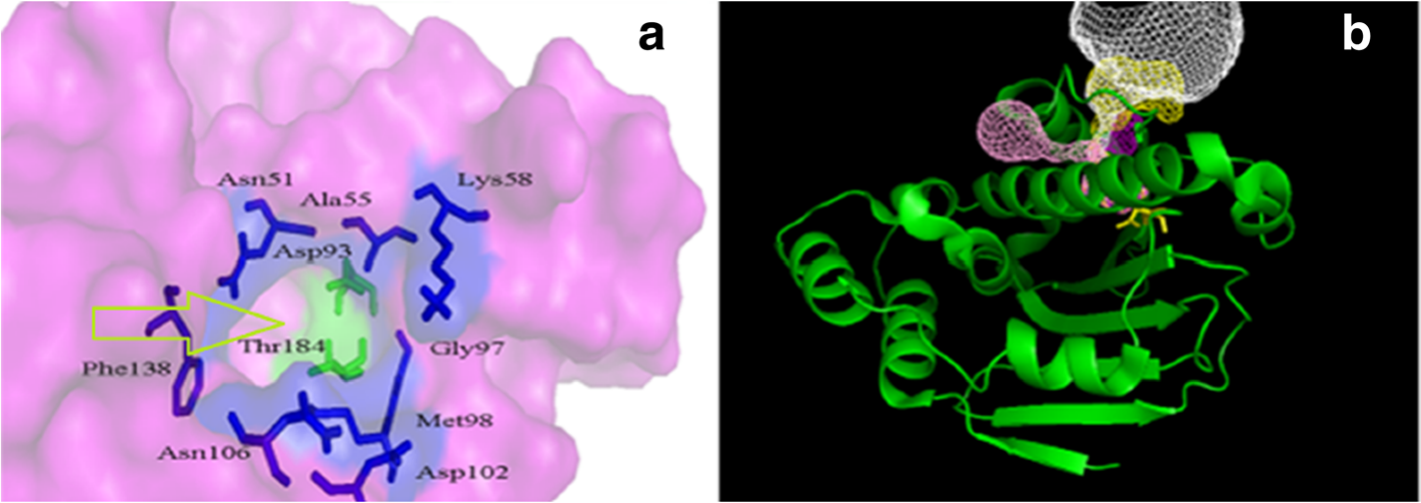
**a** Main cavity in Hsp90, **b** Four entrance tunnels in back of main cavity

As previously mentioned, Asp93 is one of the important residues in Hsp90 ATPase. The pathway into Asp93 is a large cavity, and in its crater Asn51, Ala55, Lys58, Gly97, Met98, Asp102, Asn106 and Phe138 lie. Asp93 and Thr184 stay on the bottom of the pocket (Fig. 7a). Other pathways into Asp93 and the involved residues were obtained by CAVAER 3.0.1 software [27]. Likely pathways were computed by assigning maximum probe radius to 0.9 Ǻ, shell depth to 4, and clustering threshold to 3.5 Ǻ. Four tunnels were obtained, which are depicted in Fig. 7b. As can be seen, all of the tunnels stay at the back of the cavity.All residues in the depth of the tunnel included Ile49, Ser50, Ser52, Ser53, His77, Ile78, Leu80, Ile91, Val92, Asp93, Val207, His210, Gln212, Phe213, Ile214, Tyr216, Pro217, Ile218, Thr219 and Leu220.

After the validation of the docking protocol and finding entrance tunnels, the 3D structures of 3,4-isoxazolediamide derivatives (25 compounds) were docked into crystallography structure 3OWD. In .dlg files, the structures were ranked by binding energy and clusters. All of the compounds were perched into the cavity of the active site (Table 7). The compounds were divided into three groups.

**Table 7:** Interactions between the docked 3, 4-isoxazolediamide derivatives and Hsp90 binding site residues

Comp.	IC_50_ (μM)	∆*G*_binding_ (kcal/mol)	Hydrogen bonds between atoms of compounds and amino acids		Hydrophobic amino acids
			Atom of comp.	Amino acid (Distance Å)	
15	0.153	−2.08	Ortho- OH resorcinol ring	Asp93(1.974)	Lys58, Gly135, Ala55, Ile96, Asn106, Met98, Gly97, Asn51, Ser52, Leu107, Phe138, Leu48, Thr184
			Para- OH resorcinol ring	HOH^a^(1.828)	
16	0.184	−1.90	Ortho- OH resorcinol ring	Asp93(1.910)	Lys58, Gly135, Ala55, Ile96, Asn106, Met98, Gly97, Asn51, Ser52, Leu107, Phe138, Leu48,Thr184, Val186
			Para- OH resorcinol ring	HOH(1.863)	
18	0.130	−2.15	Para- OH resorcinol ring	Lys58(2.164)	Thr152, Ile96, Ala55, Ser52, Val186, Val150, Leu107, Tyr139, Val136, Ile26, Asp93
				HOH(2.153)	
			Ortho- OH resorcinol ring	Asn106(2.155)	
			O atom- isoxazole	HOH (2.632)	
			NH- isoxazole	HOH(2.373)	
19	0.074	−2.24	Para- OH resorcinol ring	Asp93(1.887)	Lys58, Gly135, Ala55, Ile96, Asn106, Met98, Gly97, Asn51, Ser52, Leu107, Phe138, Leu48, Thr184, Val186
				HOH(2.085)	
			O atom- isoxazole	HOH(2.153)	
20	0.037	−2.85	Ortho- OH-resorcinol ring	Asp93(1.710)	Leu48, Asn51, Asp54, Ser52, Ala55,Lys58, Ile96, Met98, Leu107, Phe138, Thr184, Val186
			Para- OH-resorcinol ring	HOH(1.919)	
			NH- isoxazole	Gly97(2.373)	
21	0.085	−2.23	Para- OH-resorcinol ring	Asp93(2.044)	Leu48, Gly97, Asp54, Ser52, Ala55,Lys58, Ile96, Met98, Asn106, Leu107, Lys112, Gly135, Phe138,T hr184, Val186
			Ortho- OH-resorcinol ring	Asn51(1.970)	
22	0.167	−1.93	Ortho- OH-resorcinol ring	Asp93(1.719)	Leu48, Asn51, Asp54, Ser52, Ala55, Lys58, Ile96, Met98, Leu107, Phe138, Thr184, Val186
			Para- OH-resorcinol ring	HOH(2.085)	
			NH- isoxazole	Gly97(2.287)	
24	0.160	−1.98	Para- OH resorcinol ring	Asp93(1.819)	Leu48, Gly97, Asp54, Ser52, Ala55, Asn51, Ile96, Met98, Asn106, Leu107, Lys112, Gly135, Phe138, Thr184, Val186
				HOH(2.115)	
			O atom- isoxazole	HOH(2.200)	
			F atom-terminal ethyl	HOH(1.813)	
			O atom-methoxy	Lys58(2.105)	
26	0.055	−2.58	Para- OH-resorcinol ring	Asp93(2.225)	Leu48, Asp54, Ser52, Ala55, Asn51, Ile96, Met98, Asn106, Leu107, Phe138, Thr184, Val186
			Ortho- OH-resorcinol ring	Gly97(2.064)	
			O atom-terminal ethyl	Lys58(1.812)	
27	0.041	−2.59	Para- OH resorcinol ring	HOH(2.111)	Leu48, Gly97, Asp54, Ser52, Ala55,Lys58, Ile96, Met98, Asn51, Gly95, Asp93, Leu107, Lys112, Gly135, Phe138, Thr184, Val186
			O atom-terminal ethyl	HOH(2.459)	
			NH- terminal amid	Asn106(2.124)	
28	0.190	−1.92	Para- OH resorcinol ring	Thr184(1.724)	Asp93, Asp54, Ser52, Ala55, Asn51, Ile96, Met98, Leu107, Phe138, Val186
			Ortho- OH-resorcinol ring	Gly97(2.262)	
			NH- isoxazole	HOH(2.022)	
			NH- isoxazole	HOH(1.113)	
			NH- terminal amid	Asn106(2.492)	
30	0.240	−1.85	Para- OH-resorcinol	Ala55(1.640)	Asp93, Asp54, Ser52, Lys58, Asn51, Gly97, Met98, Asn106, Leu107, Phe138, Gly135, Thr184
			Ortho- OH-resorcinol	HOH(2.397)	
46	0.026	−3.02	Para- OH-resorcinol ring	Asp93(2.106)	Thr152, Gly97,Ile96, Val186, Ala55, Asp102, Leu107, Tyr132, Gly108, Val136, Ile110
			Ortho- OH-resorcinol ring	Asn51(2.058)	
			O atom- isoxazole	Asn51(2.805)	
			NH- isoxazole	HOH(2.433)	
			O atom-methoxy	Lys58(2.933)	
57	0.084	−2.31	Ortho- OH-resorcinol ring	Asp93(1.887)	Leu48, Asn51, Asp54, Ser52, Ala55, Lys58, Ile96, Met98, Leu107, Phe138, Thr184, Val186
			Para- OH-resorcinol ring	HOH(1.800)	
			S atom-terminal amid	HOH(1.129)	
			O atom-terminal ethyl	HOH(1.830)	
			NH- isoxazole	Gly97(2.314)	
58	0.022	−3.40	Ortho- OH-resorcinol ring	Asp93(1.799)	Leu48, Asn51, Ser52, Ala55, Lys58, Ile96, Met98, Asn106, Leu107, Phe138, Gly135, Thr184, Val186, Val150
			Para- OH-resorcinol ring	HOH(1.773)	
			NH- isoxazole	Gly97(2.375)	
59	0.250	−1.84	Para- OH-resorcinol ring	Thr184(1.814)	Asp93, Leu48,, Asn51, Ser52, Ala55, Lys58, Ile96, Met98, Gly97, Asn106, Leu107, Gly135
			O atom-terminal ethyl	HOH(2.385)	
61	0.040	−2.68	Ortho- OH-resorcinol ring	Asp93(1.832)	Leu48, Asn51, Ser52, Ala55, Lys58, Ile96, Met98, Asn106, Leu107, Phe138, Gly135, Thr184, Val186, Val150
			Para- OH-resorcinol ring	HOH(1.816)	
			NH- isoxazole	Gly97(2.328)	
			NH-thiazole	HOH(2.308)	
64	0.260	−1.82	Para- OH-resorcinol ring	Lys58(2.045)	Asp93, Asn51, Ser52, Ala55, Asp54, Ile96, Met98, Gly97, Phe138, Gly135, Val136, Tyr139, Asn106, Leu107, Thr184, Val186
				HOH(2.032)	
			Ortho- OH-resorcinol ring	Asn106(2.134)	
			O atom- isoxazole	HOH(2.449)	
			NH-isoxazole	HOH(2.340)	
65	0.048	−2.62	O atom- isoxazole	HOH(2.733)	Asp93, Asn51, Ser52, Ala55, Lys58, Ile96, Met98, Gly97, Phe138, Gly135, Val136, Tyr139, Asn106, Leu107, Thr184, Val186
66	0.062	−2.46	Para- OH-resorcinol ring	Lys58(2.144)	Asp93, Asn51, Ser52, Asp54, Ala55, Ile96, Met98, Gly97, Phe138, Gly135, Val136, Tyr139, Gly137, Leu107, Thr184, Val186
				HOH(1.937)	
			Ortho- OH-resorcinol ring	Asn106(2.074)	
			O atom- isoxazole	HOH(2.477)	
			NH- isoxazole	HOH(2.218)	
			NH-terminal ethyl	Asn106(1.829)	
69	0.020	−3.50	Para- OH-resorcinol ring	HOH(2.022)	Asn51, Ser52, Asp54, Ala55, Lys58, Ile96, Met98, Phe138, Gly135, Val136, Tyr139, Asn106, Leu107, Thr184, Val186
			Ortho- OH-resorcinol ring	Asp93(1.909)	
			S atom-terminal amid	HOH(1.969)	
			NH- isoxazole	Gly97(2.365)	
73	0.040	−2.80	Ortho- OH-resorcinol ring	Asp93(2.067)	Asn51, Ser52, Asp54, Ala55, Lys58, Ile96, Gly97, Phe138, Gly135, Val136, Gly137, Leu107, Thr184, Val186
			Para- OH-resorcinol ring	HOH(1.968)	
			NH- isoxazole	Met98(2.082)	
			O atom-terminal ethyl	HOH(2.277)	
81a	0.230	−1.88	Para- OH-resorcinol ring	Asp93(2.250)	Asn51, Ser52, Asp54, Ala55, Ile96, Met98, Asp106, Thr184
			Ortho- OH-resorcinol ring	Gly97(1.942)	
			O atom-terminal ethyl	Lys58(1.888)	
81b	0.430	−1.76	Ortho- OH-resorcinol ring	Asp93(1.704)	Lys58, Ser52, Asp54, Ala55, Ile96, Met98, Gly97, Asp106, Leu107, Thr184, Val186
			Para- OH-resorcinol ring	HOH(2.099)	
			NH- terminal methoxy	Asn51(2.160)	
81c	0.620	−1.65	Ortho- OH-resorcinol ring	Asp93(1.724)	Lys58, Ser52, Asp54, Ala55, Ile96, Met98, Asp106, Leu107, Thr184, Val186, Phe138
			Para- OH-resorcinol ring	HOH(1.860)	
			NH- isoxazole	Gly97(2.305)	
			NH-cyclohexane	Asn51(2.153)	

^a^HOH = Crystallographic water

#### Group A (compounds 15–24)

In this Group, the meta-position of resorcinol ring was substituted by a large chloro group. So, when R_1_ group was small, for example like the CH_3_ group, hydroxyl groups in resorcinol ring could interact well with Asp93. On the other hand, large groups in R_1_ such as 1,2,3-trimethoxybenzene groups, due to steric hindrance, changed steric orientation of hydroxyl groups in resorcinol and hydroxyl groups could interact with Asp93 by a water-bridge hydrogen bond. According to what was mentioned above, compound 20 was the best inhibitor in this group. Compound 18 had a 1,2,3-trimethoxybenzene group, but trimethoxy groups created a better hydrogen bond with Lys112 than methoxy group.

#### Group B (compounds 26–73)

Meta position of resorcinol ring was occupied by an isopropyl in this group. According to the biological assay, the lowest IC_50_ was related to compound 69 and it was the strongest compound that docking analysis revealed. The ortho-hydroxyl group of resorcinol ring with Asp93, para-hydroxyl with Ser52 by a water-bridge, and nitrogen atom of isoxazole with Gly97 was created the hydrogen bonds. Steric orientation in compound 58 was observed to be similar to that of 69. But, 69 was stronger than 58, because sulfur atom of R_1_ group in 69 created a hydrogen bond with Phe138 and Asn51 by a water-bridge. In compound 46, para-hydroxyl made a hydrogen bond with Asp93 and O atom in methoxy group could create H-bond with Lys58. The steric orientation in cyclohexane made compound 28, and 30 could not directly create a hydrogen bond with Asp93. The connected R_1_ group to carbonyl was isoxazole in 73 and 59 but 73 was a stronger inhibitor than 59. The connected benzene group to this isoxazole changed the steric orientation in resorcinol and could not directly make a hydrogen bond with Asp93. Unlike Group A, in this group the compounds with smaller R_1_substituent were not potent inhibitors.

#### Group C (compounds 81a-81c)

In this group, on meta-position of resorcinol ring was placed a large chloro group and R_1_ was connected to NH. 81a was stronger than both 81b and 81c. Thiophene group was better than either methoxybenzen or cyclohexane. The same as Group A, the compounds with small R_1_ groups were stronger inhibitors.

### Predicted ligands and molecular dynamic simulation

**Fig. 8:**
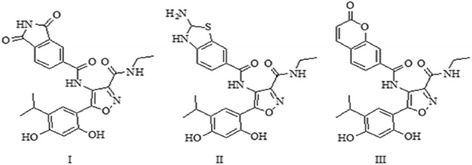
The predicted novel inhibitors based on QSAR model and docking

Three novel compounds (Fig. 8) were predicted with the modification of compounds based on results of QSAR model and molecular docking.

**Fig. 9:**
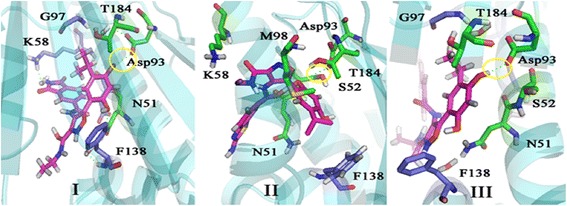
Orientation and main interaction between three predicted compounds and Hsp90

Docking study was performed on the predicted compounds. In these compounds, similar to previously evaluated compound, the hydroxyl groups of resorcinol ring interacted with Asp93 (Fig. 9). The binding energy of compound II was the lowest (−8.20 kcal/mol).

To ensure the stability of the new ligand II in the active site of protein, the MD simulation was carried out and its interaction modes with compound 69 in Hsp90 active site was also compared. The best conformation of docking was chosen to perform 50 ns MD simulation. The time-dependent behavior of MD trajectories were analyzed including root mean square deviation (RMSD) for all backbone atoms and ligands, average fluctuations of the residues (RMSF) and gyration radius (Rg) for all backbone atoms.

**Fig. 10:**
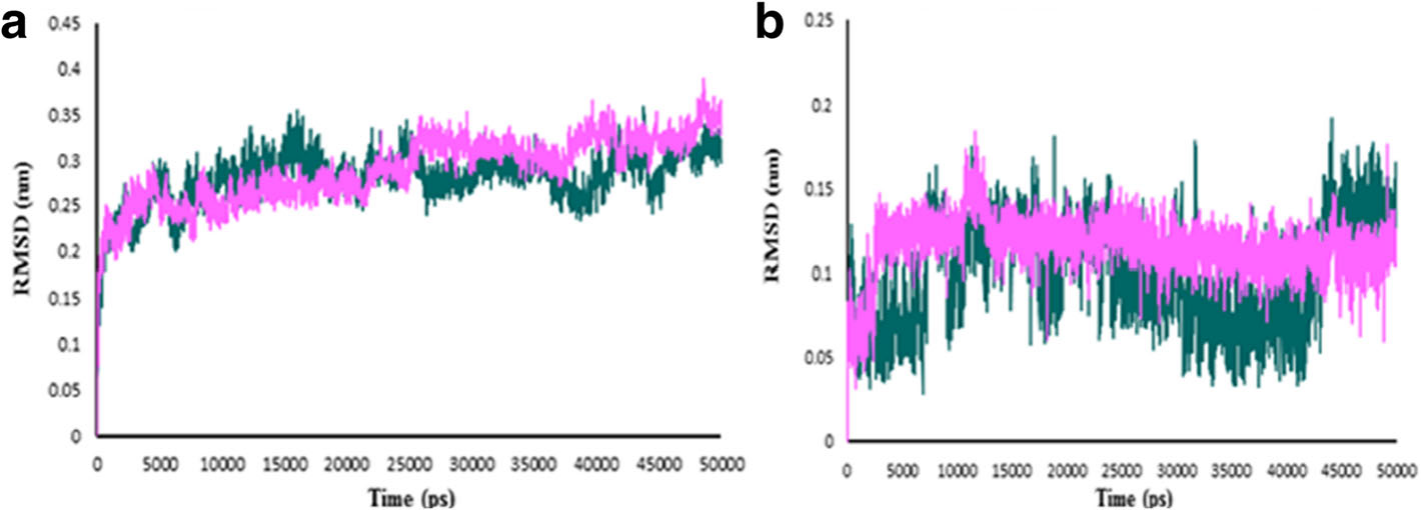
The RMSD profile. **a** Hsp90 backbone in complex with compound 69 (*violet*), predicted ligand II (*green*). **b** Compound 69 (*violet*), predicted ligand II (*green*) as a function of simulation time

**Fig. 11:**
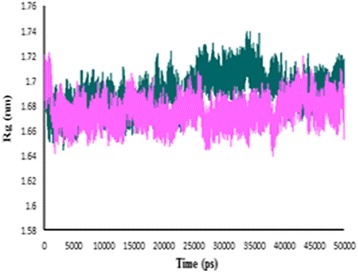
The gyration radius plot of backbone. Compound 69 (*violet*), predicted ligand II (*green*)

The RMSD of backbone atoms was figured to assessment the conformational stability of the protein during the simulation. The RMSD of backbone atoms between complexes of Hsp90-ligand 69 and Hsp90-predicted ligand II was measured with a 10 ps time interval. As illustrated in Fig. 10a, the RMSD profile in the two complexes was almost the same in the first 10 ns. Variations of RMSD were not very considerable, which denote the stability of both complexes. The ligand RMSD profile in Fig. 10b shows that the ligand 69 after 3 ns and predicted ligand II after 8 ns become stable. The RMSD variations were almost 0.1 nm in ligand 69 and predicted ligand II, it can be observed that both of the ligands were also fit in the active site and stabilized. The protein compactness was evaluated by the gyration radius (Rg). The Rg plot is shown in Fig. 11. In the first 27 ns and the last 10 ns, the Rg values of ligand 69 and the predicted ligand II were superimposed and the conjunction of both complexes was conserved during the simulation.

**Fig. 12:**
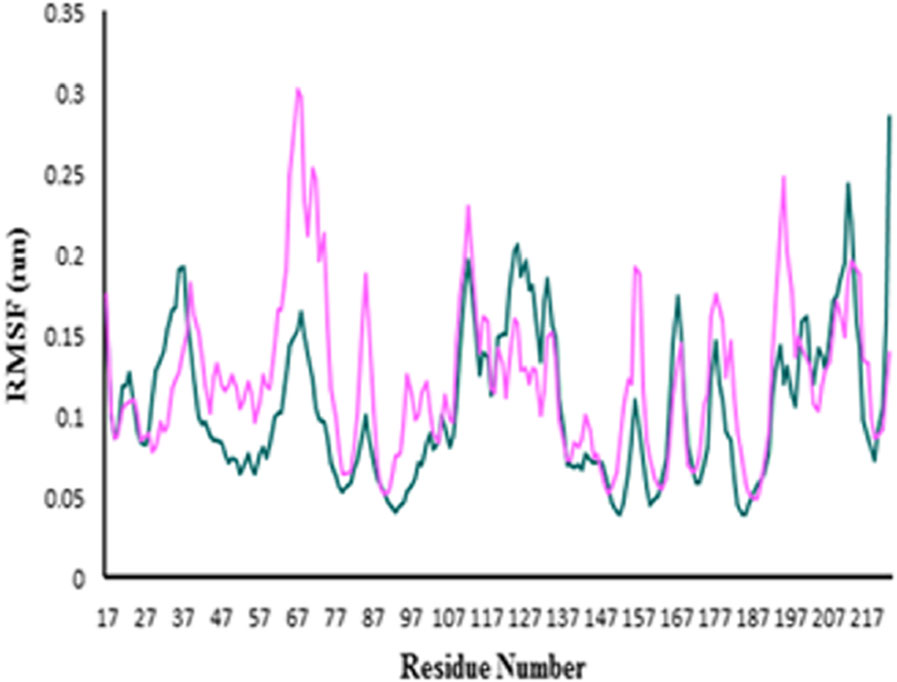
The RMSF plot. Hsp90-compound 69 (*violet*), Hsp90-predicted ligand II (*green*)

The variations of protein flexibility were identified by the root mean square fluctuation (RMSF) of backbone residues. As shown in Fig. 12, in residues 45–61 and 89–107, the fluctuation of predicted ligand II was lower than compound 69 which disclose that predicted ligand II was more stable than compound 69 in these parts. This can be attributed to the attendance of these residues in the active site of protein. In residues 61–70 the fluctuation was high, which indicates that these parts of the protein were more unstable than other parts during the MD simulation, especially in the predicted ligand II.

**Fig. 13:**
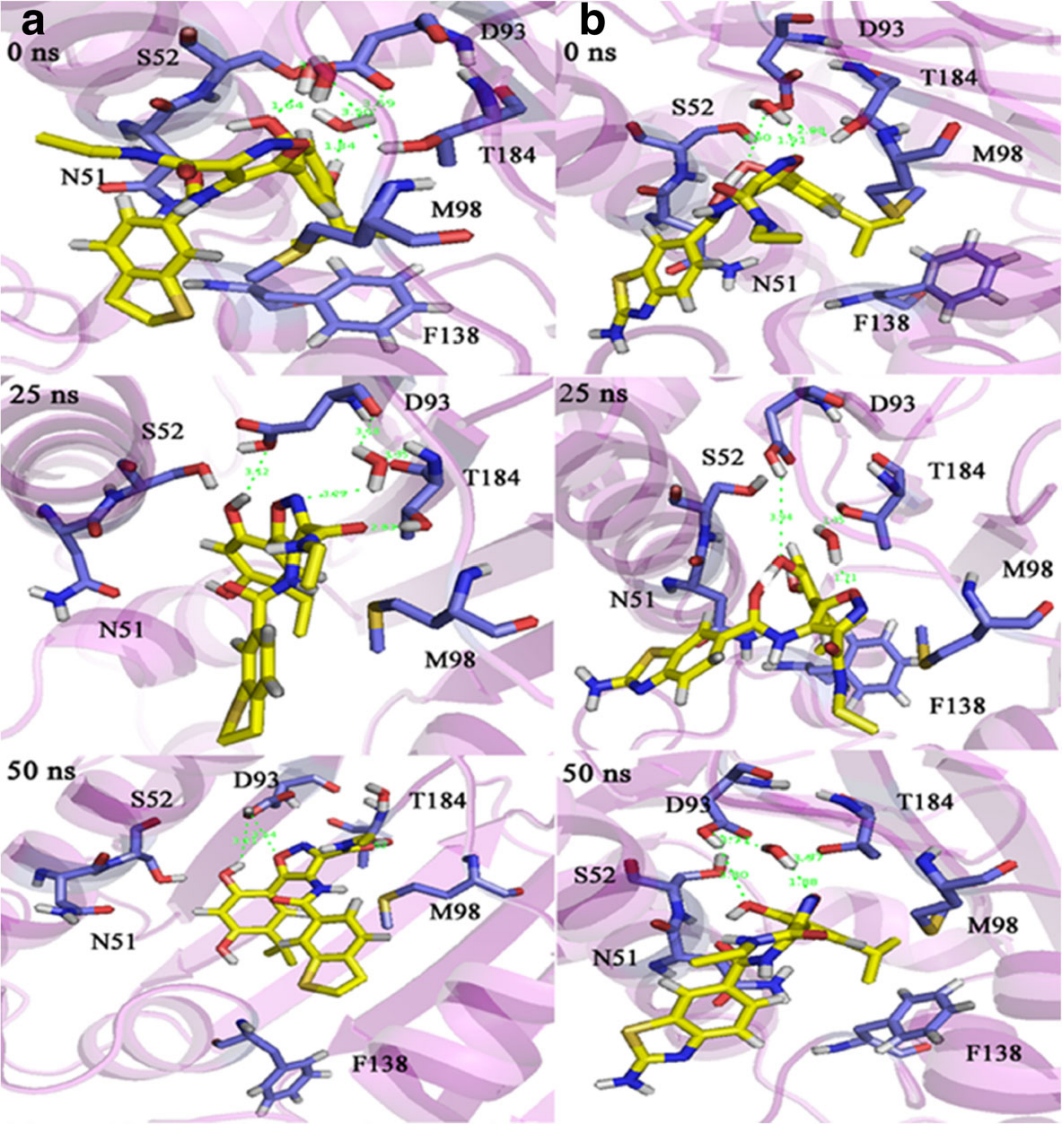
3D plots of the interaction between two ligands and Hsp90 at different times during the MD simulation. Column **a** The interaction of compound 69 with Hsp90. Column **b** the interaction of the predicted ligand II with Hsp90

To discover the conformations and interactions of the predicted ligand II and compound 69 during simulation, 3D plots of the different times of simulation were shown (Fig. 13). Figure 13 shows the comparison between the compound 69 and predicted ligand II in the active site of Hsp90 during 0, 25 and 50 ns of the simulation (a, b columns). In all simulation times, the resorcinol hydroxyl group of both compounds formed hydrogen bond with Asp93. On other side of both compounds perched toward out of pocket and was not involved in interactions between ligand and protein. Since the beginning until the half of simulation (0–25 ns), the hydrogen bond was built with crystallography water molecules in both compounds. In compound 69, at the end of simulation (50 ns), the N atom of isoxazole ring and the resorcinol hydroxyl group made hydrogen bonds with Asp93 but the crystallography water molecules was not involved in interactions between ligand and protein. Adding amine group to benzothiophene moiety, conformation of predicted ligand changed and the interaction between ligand and the crystallography water molecule was seen with 1.88 Å distance. According to the MD simulation analysis and also the evaluation of the interactions between compound 69 and predicted ligand II with Hsp90, adding amine group was effective in improving of binding energy of ligand and protein.

## Conclusion

In current study, quantitative relationships between the molecular structure and Hsp90 inhibitory activity of 3, 4-isoxazolediamide derivative were verified by MLR and GA-PLS. In MLR, the connectivity index (X5A) has a significant effect on the inhibitory activity. This method made suitable results for the prediction set. In addition to MLR, genetic algorithm method was used which illustrated much more promising results in comparison with stepwise regression. It can be concluded from the two methods that structural features of the molecule such as size, shape, symmetry, and branching are particularly important in design of new Hsp90 inhibitors based on 3,4-isoxazolediamide scaffold. The molecular docking simulation was also done for 25 compounds in three groups (A, B and C).

Based on QSAR models and molecular docking analysis were predicted three novel compounds. The ligand II was chosen in term of binding energy and steric orientation. Molecular dynamic simulation was done and analyses such as RMSD, RMSF and Rg, displayed that proposed ligand II, is stable in the Hsp90 active sites.

## Abbreviations

ATP: Adenosine triphosphate
CDFS: Combined data splitting-features election
GA-PLS: Genetic algorithm partial least squares
Hsp90: Heat shock protein90
MDs: Molecular dynamic simulation
MLR: Multiple linear regression
Q^2^_LOO_: Square correlation coefficient for leave-one-out cross-validation
QSAR: Quantitative structure activity relationship
R^2^c: Calibration correlation coefficient
R^2^_p_: Prediction correlation coefficient
Rg: Gyration radius
RMS_CV_: Root mean square error of cross-validation
RMSF: Root mean square fluctuation
RSMD: Root-mean-square deviation
S.E: Standard error of calibration
